# Supplementary material for: Immersive scene representation in human visual cortex with ultra-wide-angle neuroimaging
Source: Nat Commun. 2024 Jun 28;15:5477. doi: 10.1038/s41467-024-49669-0 (PMC11213904; doi:10.1038/s41467-024-49669-0)
Supplement: Supplementary file 1 — Supplementary Information [file 41467_2024_49669_MOESM1_ESM.pdf]

Supplementary Information

Immersive scene representation in human visual cortex with ultra-wide angle neuroimaging

Jeongho Park[1,*], Edward Soucy[2], Jennifer Segawa[2], Ross Mair[2,3,4] and Talia Konkle[1,2,5]

[1]*Department of Psychology, Harvard University, Cambridge, MA, USA*
[2]*Center for Brain Science, Harvard University, Cambridge, MA, USA*
[3]*Department of Radiology, Harvard Medical School, Boston, MA, USA*
[4]*Department of Radiology, Massachusetts General Hospital, Boston, MA, USA*
[5]*Kempner Institute for Biological and Artificial Intelligence, Harvard University, Boston, MA, USA*
[*]*email: jpark3@g.harvard.edu*

Signal Quality Assessment

To compare the MRI signal quality, we ran the same protocol twice, with and without the top head-coil. Specifically, we examined impacts of no head-coil on the anatomical image (MPRAGE) and the functional data (BOLD). First, we ran a special sequence designed for quality assessment of the head coils. It produced pure image SNR maps from each of principal axes (Fig. S1a), which is nominally signal divided by the noise. Qualitatively, the image SNR in the posterior part of brain was barely affected, and the resulting T1-weighted images confirmed this (Fig. S1b).

Next, we ran one of our BOLD scans (functional localizer) with the exact parameters to get tSNR, which is important for BOLD sensitivity as it shows an ability to detect changes during the time course of the scan. The acquired BOLD data were preprocessed, brain-masked, then the mean and standard deviation images were calculated. The tSNR image was obtained from dividing the mean by standard deviation. To compare only brain areas acquired predominantly by the bottom head-coil only, we created a mask. In the bottom head-coil only tSNR image, we selected areas above the chosen threshold (tSNR = 25), then masked the with top head-coil tSNR image with the selection. The resulting average tSNR values were 34.9 without the top head-coil and 48.1 with the top head-coil. As reference, the tSNR images are shown in Fig. S1c.

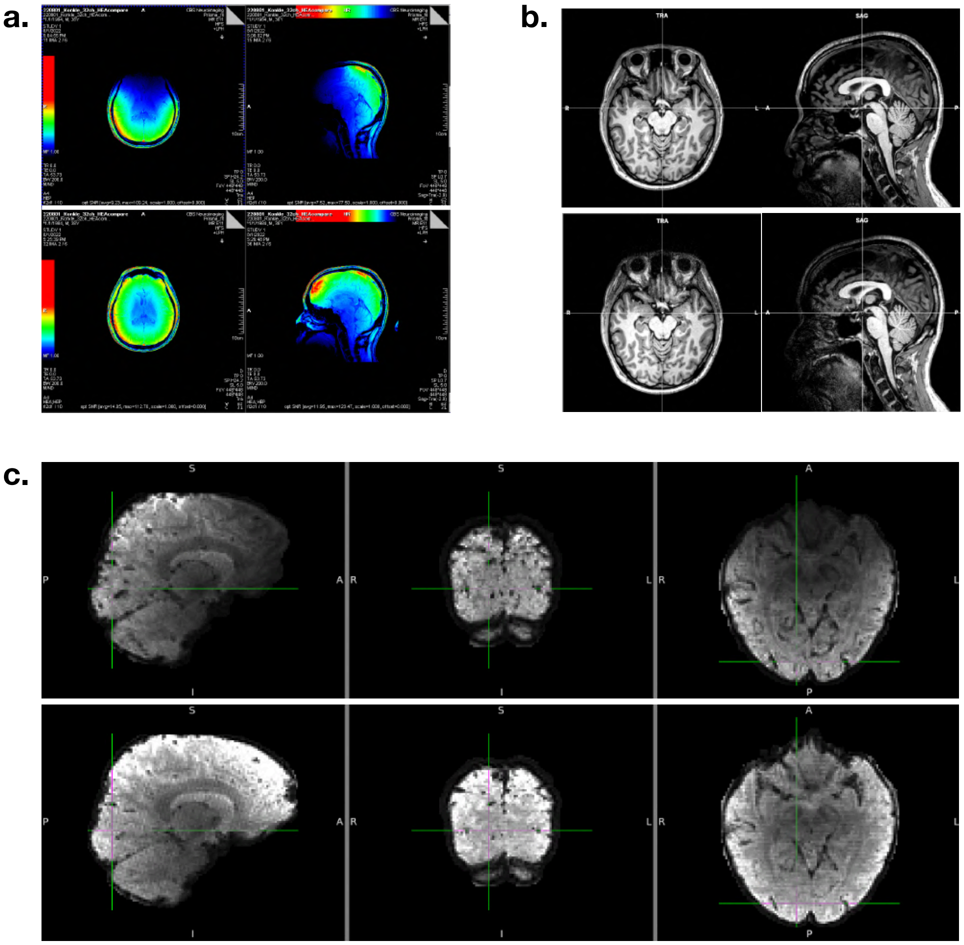

Supplementary Figure 1: *(A) Single slice SNR maps (horizontal, sagittal), with (bottom) and without the top head-coil (top). (B) Constructed T1-weighted image. (C) tSNR Images, with (bottom) and without the top head-coil (top).*

Lastly, to provide a direct assessment of signal quality in the regions-of-interest (ROIs), we quantified the tSNR within each ROI. For this, we preprocessed the data using the same protocol as the main experiments and normalized it into Talairach space. The voxel-wise tSNR was calculated by dividing the mean by the standard deviation of timecourse data. Then, we extracted voxels for each ROI, and averaged their tSNRs to get an ROI tSNR value. The comparison between with and without the top head-coil is reported in Fig. S2. There was clear decrease of tSNR in all of the main ROIs when the top head-coil was removed. However, overall tSNR values even without the top head-coil are in the high range (>100), due to the well-powered experimental design, and other standard pre-processing practices like spatial smoothing, re-slicing, and averaging across voxels within an ROI, which are the identical steps we took for the actual ROI analyses.

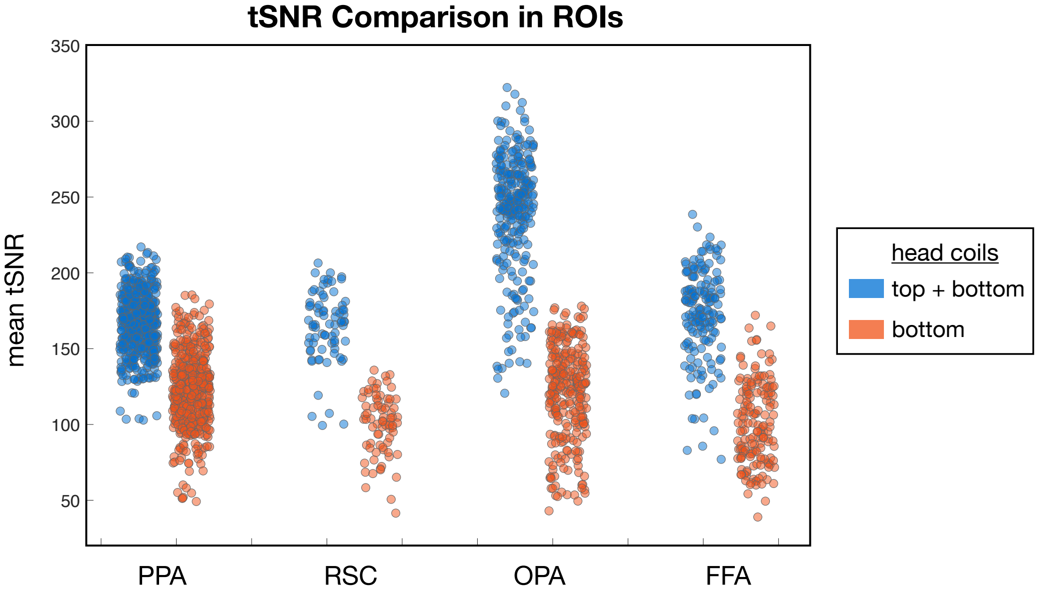

Supplementary Figure 2: *Comparison of tSNR in Regions of Interests (ROIs). PPA: Parahippocampal Place Area (n=479 voxels), RSC: Retrosplenial Cortex (n=76 voxels), OPA: Occipital Place Area (n=271 voxels), FFA: Fusiform Face Area (n=158 voxels).*

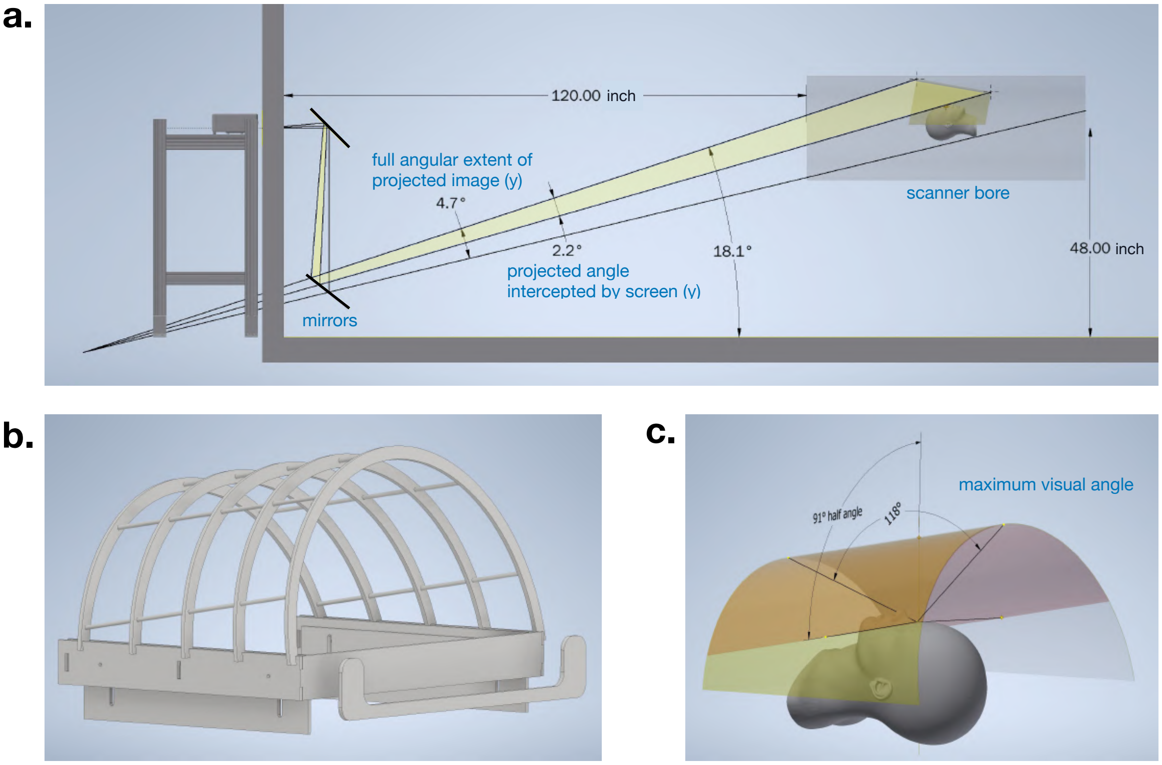

Supplementary Figure 3: *(A) The geometry of MRI room and image projection path. (B) Screen Hull. (C) Maximum visual angle.*

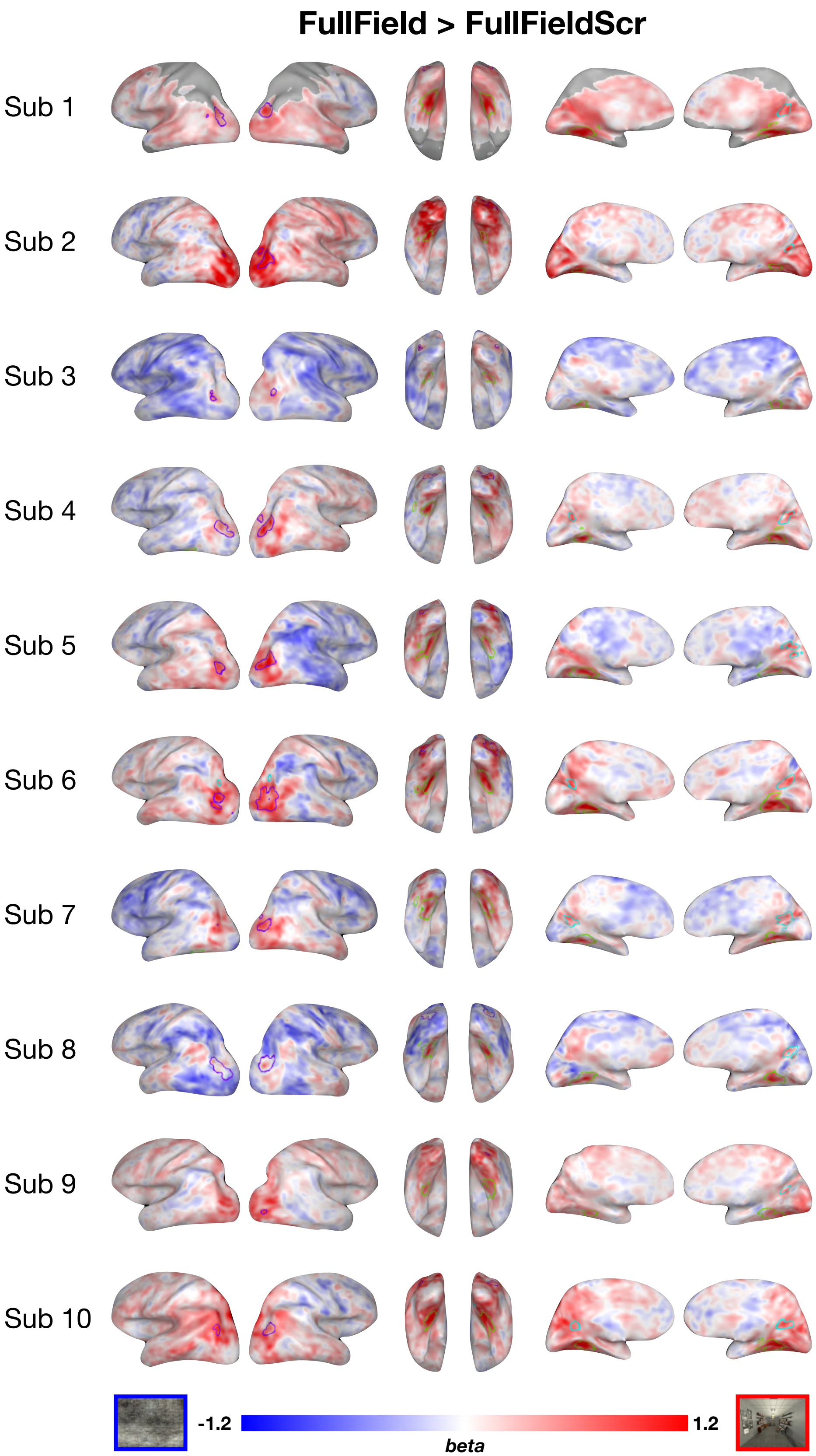

Supplementary Figure 4: *Exp2 Whole brain contrast map (Full-field scenes - Phase-scrambled scenes).*

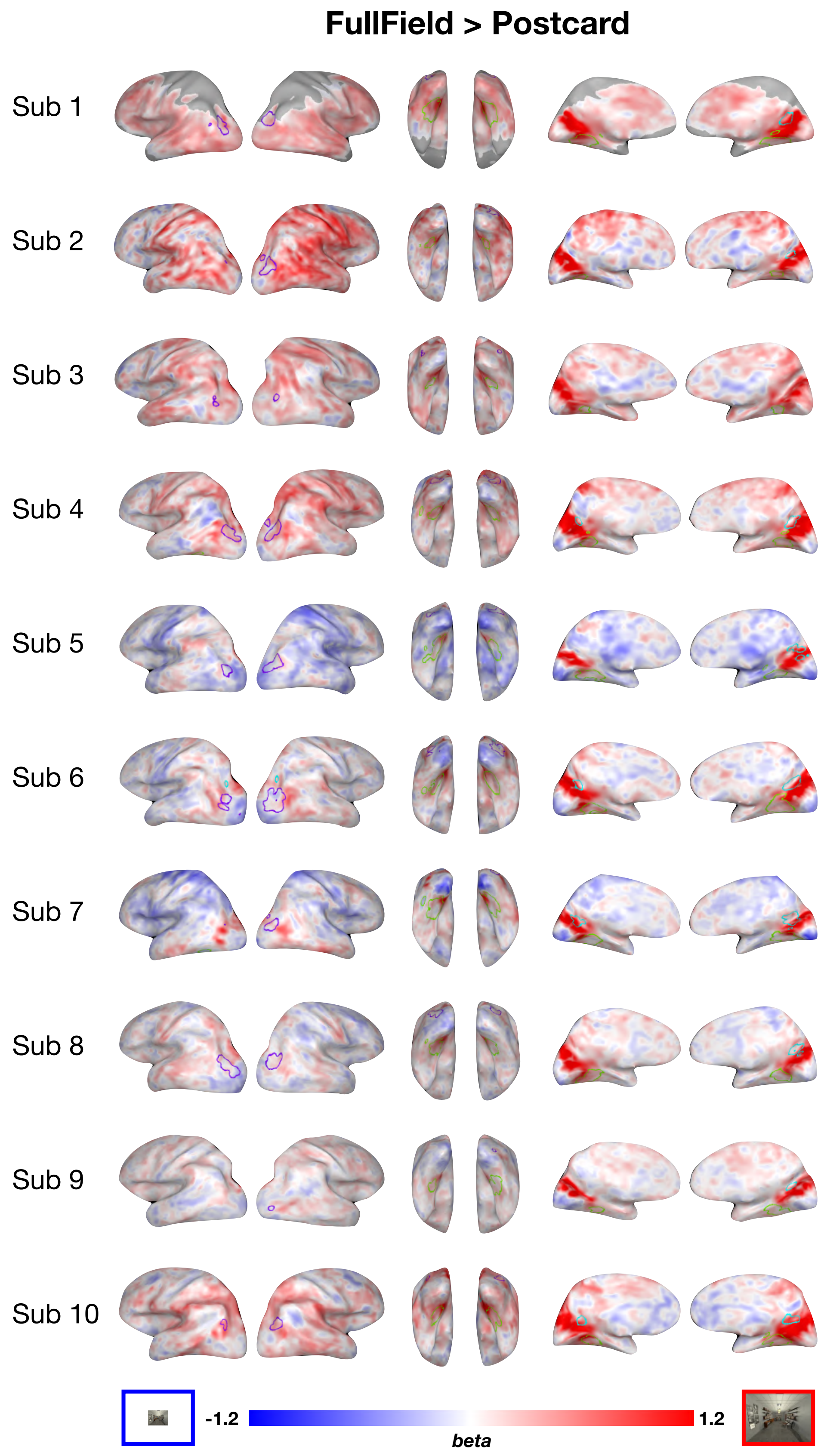

Supplementary Figure 5: *Exp2 Whole brain contrast map (Full-field scenes - Postcard scenes).*

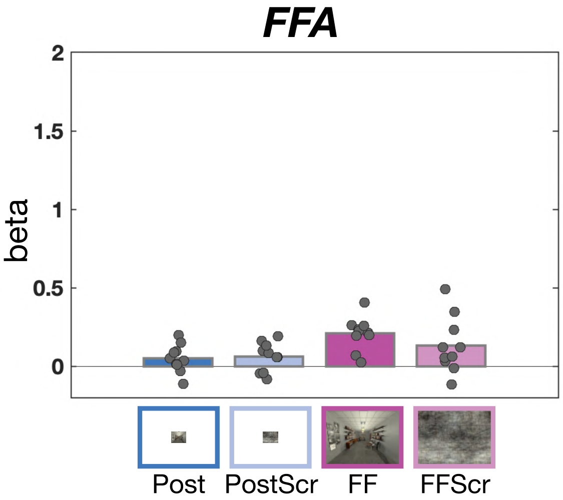

Supplementary Figure 6: *FFA ROI anlaysis (n=10). While the overall responses to all four conditions were quite low, there was a small but statistically reliable main effect of visual size, with higher overall activation in full-field over postcard views (Post: Postcard, PostScr: Postcard Scrambled, FF: Full-Field Scenes, FFscr: Full-Field Scenes Scrambled, FFA: Fusiform Face Area).*

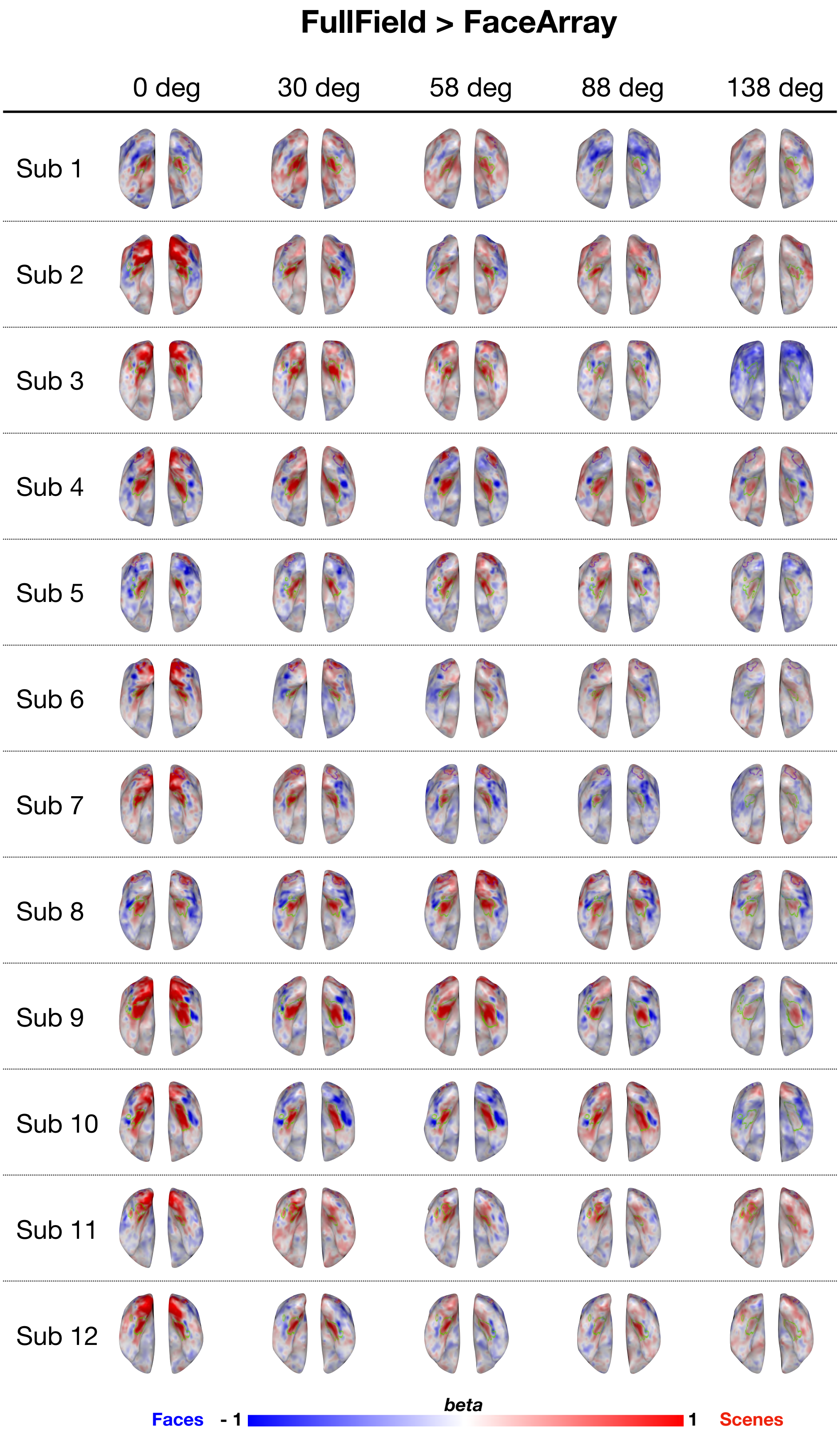
FullField > FaceArray
0 deg
30 deg
58 deg
88 deg
138 deg
Sub 1
Sub 2
Sub 3
Sub 4
Sub 5
Sub 6
Sub 7
Sub 8
Sub 9
Sub 10
Sub 11
Sub 12
beta
Faces
- 1
1
Scenes

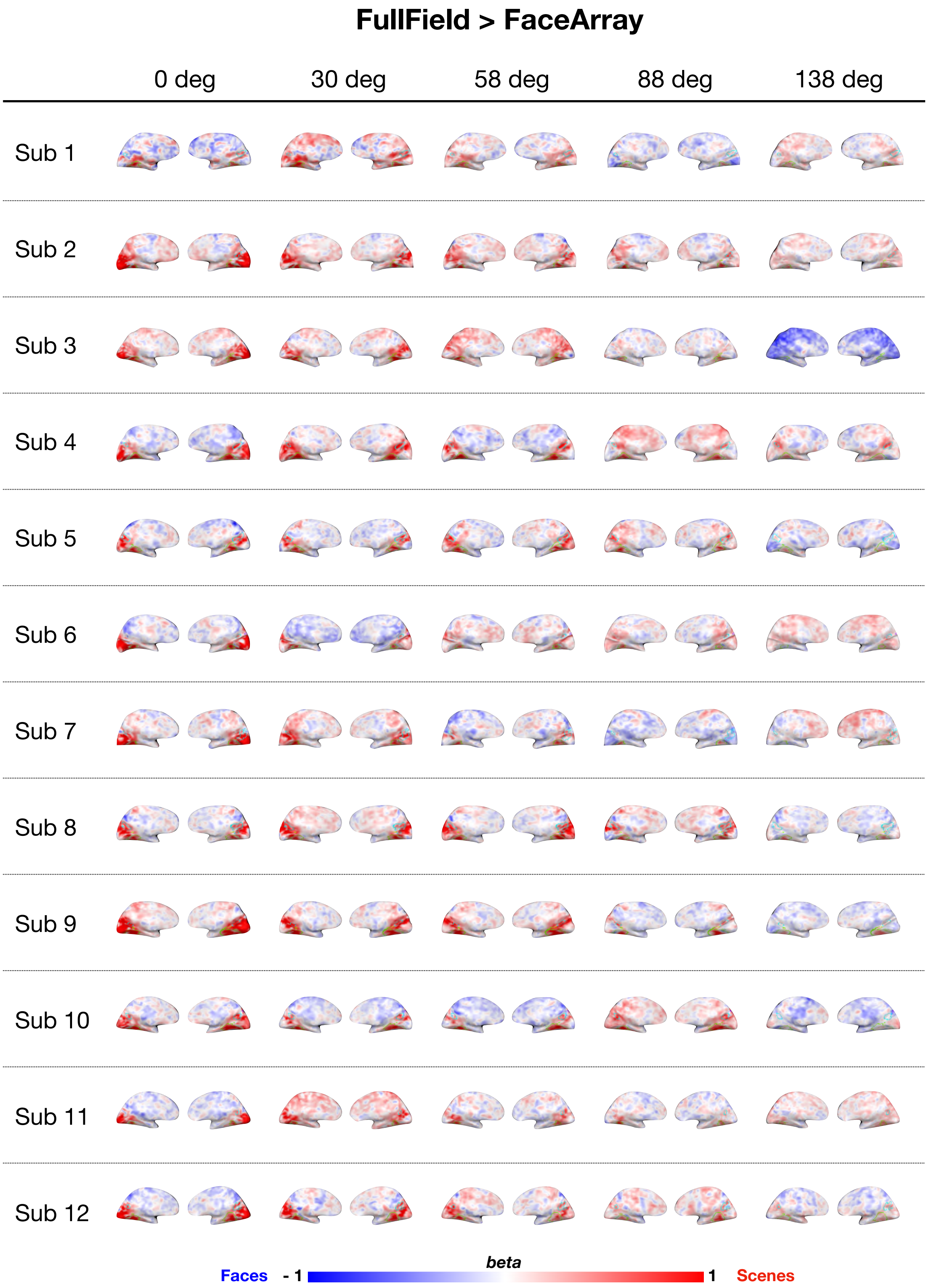
FullField > FaceArray
0 deg
30 deg
58 deg
88 deg
138 deg
Sub 1
Sub 2
Sub 3
Sub 4
Sub 5
Sub 6
Sub 7
Sub 8
Sub 9
Sub 10
Sub 11
Sub 12
beta
Faces
- 1
1
Scenes

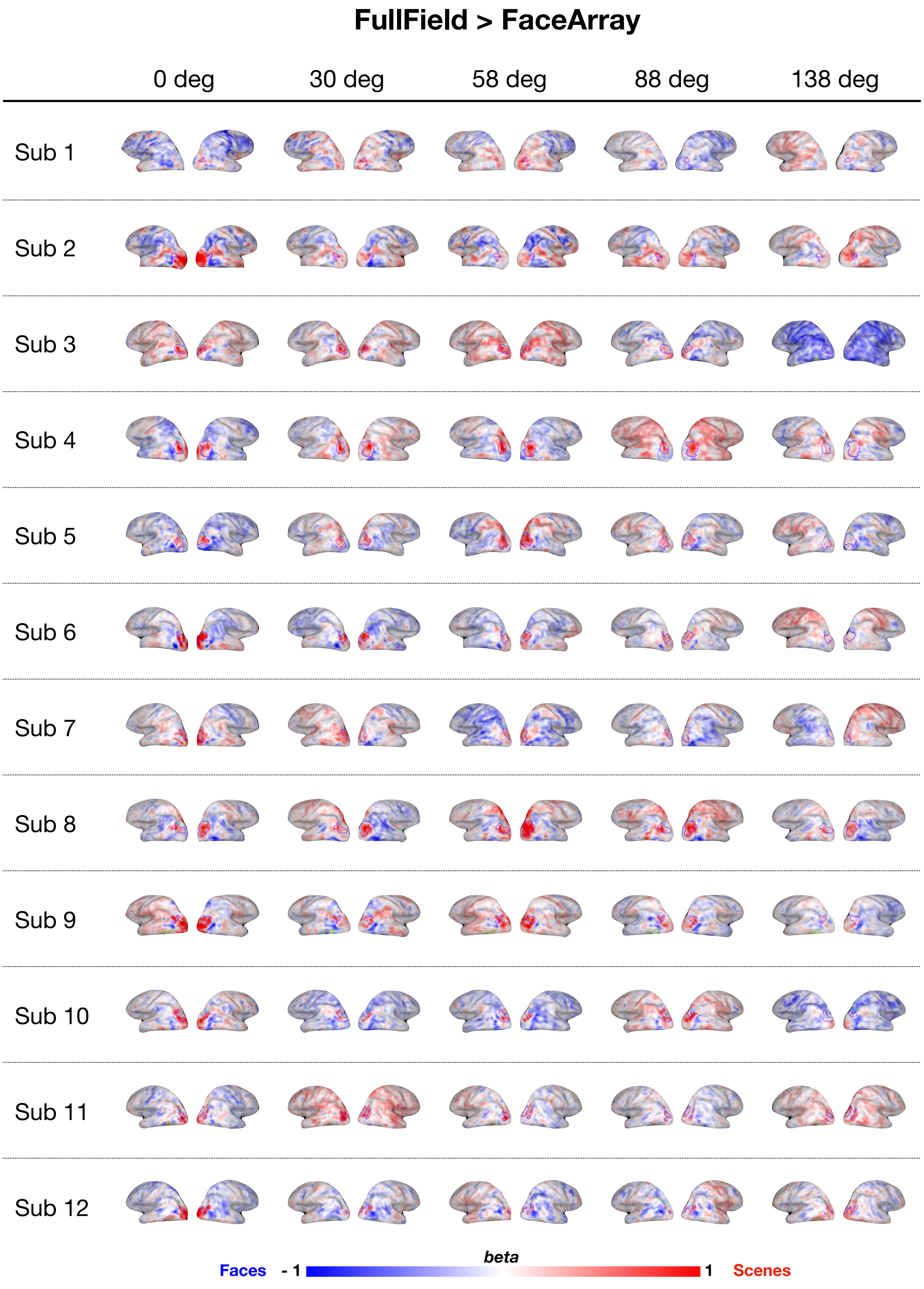

Supplementary Figure 7: *Exp3 Whole brain contrast map (Full-field scenes - Face arrays), at each scotoma level.*

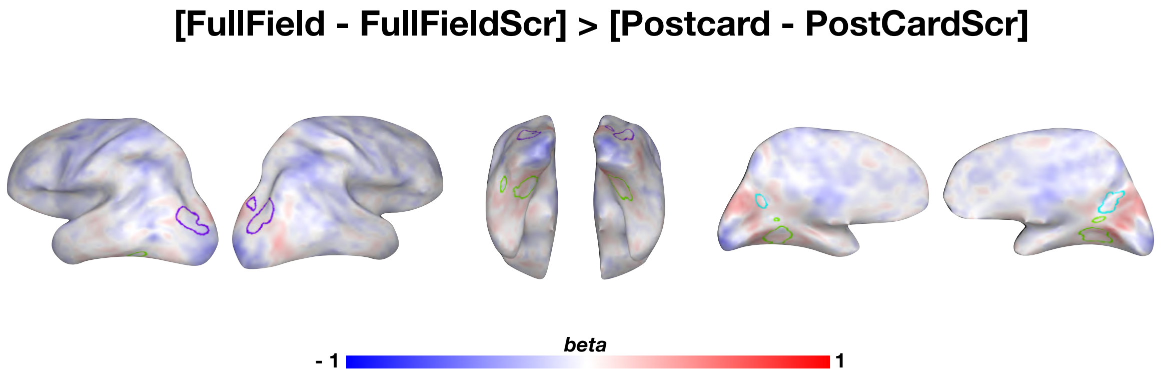

Supplementary Figure 8: *Exp2. Conjunction Contrast Map (group). In addition to the contrasts reported in the main text, we also directly ran a conjunction analysis. We only found very weak response differences in the Peripheral-POS.*

Table S1. **PPA**

	0 deg	30 deg	58 deg	88 deg	138 deg
**Scene vs Face**	t(9) = 19.95, *p*< 0.0001	t(9) = 23.94, *p*< 0.0001	t(9) = 18.76, *p*< 0.0001	t(9) = 10.76, *p*< 0.0001	t(9) = 2.65, *p*=0.0266
**Scene vs Scrambled**	t(9) = 14.22, *p*< 0.0001	t(9) = 14.7, *p*< 0.0001	t(9) = 12.05, *p*< 0.0001	t(9) = 5.89, *p*=0.0002	t(9) = 2.65, *p*=0.0264
**Scene vs Object**	t(9) = 11.28, *p*< 0.0001	t(9) = 11.38, *p*< 0.0001	t(9) = 11.92, *p*< 0.0001	t(9) = 7.81, *p*< 0.0001	t(9) = 2.13, *p*=0.0623
**Face vs Object**	t(9) = -5.38, *p*=0.0004	t(9) = -8.62, *p*< 0.0001	t(9) = -7.51, *p*< 0.0001	t(9) = -5.72, *p*=0.0003	t(9) = -2.12, *p*=0.0628
**Face vs Scrambled**	t(9) = -4.29, *p*=0.002	t(9) = -4.54, *p*=0.0014	t(9) = -5.06, *p*=0.0007	t(9) = -4.55, *p*=0.0014	t(9) = -1.63, *p*=0.1374
**Scrambled vs Object**	t(9) = -0.95, *p*=0.3677	t(9) = -1.68, *p*=0.1271	t(9) = -0.43, *p*=0.6786	t(9) = -1.92, *p*=0.0874	t(9) = -1.91, *p*=0.0884

Table S2. **RSC**

	0 deg	30 deg	58 deg	88 deg	138 deg
**Scene vs Face**	t(8) = 11.64, *p*< 0.0001	t(8) = 11.83, *p*< 0.0001	t(8) = 11.73, *p*< 0.0001	t(8) = 6.48, *p*=0.0002	t(8) = 3.57, *p*=0.0073
**Scene vs Scrambled**	t(8) = 10.34, *p*< 0.0001	t(8) = 9.99, *p*< 0.0001	t(8) = 7.43, *p*=0.0001	t(8) = 3.92, *p*=0.0044	t(8) = 3.29, *p*=0.011
**Scene vs Object**	t(8) = 12.97, *p*< 0.0001	t(8) = 9.41, *p*< 0.0001	t(8) = 12.54, *p*< 0.0001	t(8) = 6.84, *p*=0.0001	t(8) = 3.88, *p*=0.0047
**Face vs Object**	t(8) = 0.08, *p*=0.9352	t(8) = 0.25, *p*=0.8068	t(8) = -1.78, *p*=0.1134	t(8) = -2.55, *p*=0.0341	t(8) = -1.26, *p*=0.2423
**Face vs Scrambled**	t(8) = -1.28, *p*=0.2359	t(8) = -2.13, *p*=0.0661	t(8) = -2.2, *p*=0.0592	t(8) = -3.21, *p*=0.0124	t(8) = -0.45, *p*=0.6656
**Scrambled vs Object**	t(8) = 1.06, *p*=0.3204	t(8) = 2.43, *p*=0.0413	t(8) = 1.1, *p*=0.3028	t(8) = 1.04, *p*=0.3271	t(8) = -1, *p*=0.3481

Table S3. **OPA**

	0 deg	30 deg	58 deg	88 deg	138 deg
**Scene vs Face**	t(9) = 10.14, *p*< 0.0001	t(9) = 15.29, *p*< 0.0001	t(9) = 13.01, *p*< 0.0001	t(9) = 10.92, *p*< 0.0001	t(9) = 1.68, *p*=0.1273
**Scene vs Scrambled**	t(9) = 8.78, *p*< 0.0001	t(9) = 10.09, *p*< 0.0001	t(9) = 9.16, *p*< 0.0001	t(9) = 5.56, *p*=0.0004	t(9) = 2.09, *p*=0.0659
**Scene vs Object**	t(9) = 6.14, *p*=0.0002	t(9) = 8.56, *p*< 0.0001	t(9) = 13.6, *p*< 0.0001	t(9) = 6.7, *p*=0.0001	t(9) = 0.65, *p*=0.5346
**Face vs Object**	t(9) = -1.99, *p*=0.0778	t(9) = -2.58, *p*=0.0296	t(9) = -3.98, *p*=0.0032	t(9) = -4.21, *p*=0.0023	t(9) = -1.68, *p*=0.1277
**Face vs Scrambled**	t(9) = -0.35, *p*=0.7345	t(9) = -0.86, *p*=0.4108	t(9) = -3.15, *p*=0.0118	t(9) = -2.15, *p*=0.0604	t(9) = 0.4, *p*=0.6959
**Scrambled vs Object**	t(9) = -0.96, *p*=0.3601	t(9) = -1.35, *p*=0.2098	t(9) = -0.03, *p*=0.9755	t(9) = -1.05, *p*=0.319	t(9) = -2.86, *p*=0.0188

Table S4. **FFA**

	0 deg	30 deg	58 deg	88 deg	138 deg
**Scene vs Face**	t(9) = -10.61, *p*< 0.0001	t(9) = -9.16, *p*< 0.0001	t(9) = -8.54, *p*< 0.0001	t(9) = -10.22, *p*< 0.0001	t(9) = -5.16, *p*=0.0006
**Scene vs Scrambled**	t(9) = 7.08, *p*=0.0001	t(9) = 3.75, *p*=0.0045	t(9) = 0.32, *p*=0.757	t(9) = 0.83, *p*=0.4295	t(9) = 1.17, *p*=0.2733
**Scene vs Object**	t(9) = -1.37, *p*=0.2034	t(9) = -6.56, *p*=0.0001	t(9) = -4.7, *p*=0.0011	t(9) = -4.21, *p*=0.0023	t(9) = -2.54, *p*=0.0319
**Face vs Object**	t(9) = 13.16, *p*< 0.0001	t(9) = 6.68, *p*=0.0001	t(9) = 6.87, *p*=0.0001	t(9) = 8.43, *p*< 0.0001	t(9) = 3.4, *p*=0.0079
**Face vs Scrambled**	t(9) = 14.24, *p*< 0.0001	t(9) = 9.08, *p*< 0.0001	t(9) = 8.69, *p*< 0.0001	t(9) = 9.1, *p*< 0.0001	t(9) = 8.14, *p*< 0.0001
**Scrambled vs Object**	t(9) = -4.18, *p*=0.0024	t(9) = -6.79, *p*=0.0001	t(9) = -4.16, *p*=0.0024	t(9) = -3.85, *p*=0.0039	t(9) = -2.55, *p*=0.0313

Supplementary Table. Statistical results of the two-sided pairwise t-test between each image content pair, for each ROI.
