# Supplementary material for: Immersive scene representation in human visual cortex with ultra-wide-angle neuroimaging
Source: Nat Commun. 2024 Jun 28;15:5477. doi: 10.1038/s41467-024-49669-0 (PMC11213904; doi:10.1038/s41467-024-49669-0)
Supplement: Supplementary file 3 — Reporting Summary [file 41467_2024_49669_MOESM3_ESM.pdf]

nature portfolio

Corresponding author(s): Jeongho Park

Last updated by author(s): May 15, 2024

# Reporting Summary

Nature Portfolio wishes to improve the reproducibility of the work that we publish. This form provides structure for consistency and transparency in reporting. For further information on Nature Portfolio policies, see our Editorial Policies and the Editorial Policy Checklist.

## Statistics

For all statistical analyses, confirm that the following items are present in the figure legend, table legend, main text, or Methods section.

| n/a | Confirmed | |
|---|---|---|
| ☐ | ☒ | The exact sample size ($n$) for each experimental group/condition, given as a discrete number and unit of measurement |
| ☐ | ☒ | A statement on whether measurements were taken from distinct samples or whether the same sample was measured repeatedly |
| ☐ | ☒ | The statistical test(s) used AND whether they are one- or two-sided<br>*Only common tests should be described solely by name; describe more complex techniques in the Methods section.* |
| ☒ | ☐ | A description of all covariates tested |
| ☐ | ☒ | A description of any assumptions or corrections, such as tests of normality and adjustment for multiple comparisons |
| ☐ | ☒ | A full description of the statistical parameters including central tendency (e.g. means) or other basic estimates (e.g. regression coefficient) AND variation (e.g. standard deviation) or associated estimates of uncertainty (e.g. confidence intervals) |
| ☐ | ☒ | For null hypothesis testing, the test statistic (e.g. $F$, $t$, $r$) with confidence intervals, effect sizes, degrees of freedom and $P$ value noted<br>*Give P values as exact values whenever suitable.* |
| ☒ | ☐ | For Bayesian analysis, information on the choice of priors and Markov chain Monte Carlo settings |
| ☒ | ☐ | For hierarchical and complex designs, identification of the appropriate level for tests and full reporting of outcomes |
| ☒ | ☐ | Estimates of effect sizes (e.g. Cohen's $d$, Pearson's $r$), indicating how they were calculated |

*Our web collection on statistics for biologists contains articles on many of the points above.*

## Software and code

Policy information about availability of computer code

| | |
|---|---|
| Data collection | All stimuli presentation and the experiment program were produced and controlled by MATLAB R2020b (9.9.0.1592791) and Psychophysics Toolbox (3.0.17). Raw MRI images were collected using commercial Siemens software. |
| Data analysis | The fMRI data were analyzed with commercial software BrainVoyager (21.2.0), BrainVoyager (QX 2.8) with custom code written in MATLAB R2018b, and open-source Freesurfer software. |

For manuscripts utilizing custom algorithms or software that are central to the research but not yet described in published literature, software must be made available to editors and reviewers. We strongly encourage code deposition in a community repository (e.g. GitHub). See the Nature Portfolio guidelines for submitting code & software for further information.

## Data

Policy information about availability of data

All manuscripts must include a data availability statement. This statement should provide the following information, where applicable:

- Accession codes, unique identifiers, or web links for publicly available datasets
- A description of any restrictions on data availability
- For clinical datasets or third party data, please ensure that the statement adheres to our policy

Data for this project are available at the Open Science Framework repository ( https://osf.io/5hsbv/).

## Research involving human participants, their data, or biological material

Policy information about studies with human participants or human data. See also policy information about sex, gender (identity/presentation), and sexual orientation and race, ethnicity and racism.

| | |
|---|---|
| Reporting on sex and gender | Twenty two participants were recruited, and 10 of them identified their gender as female (self-report). The sex and gender were not considered in the study design, and none of analyses were sex- or gender-based. |
| Reporting on race, ethnicity, or other socially relevant groupings | N/A |
| Population characteristics | see below |
| Recruitment | Participants were recruited without any specific selection criteria besides the following: not claustrophobic, able to remain still during fMRI experiments, no metal in their bodies, and normal (or corrected-to- normal) vision. It is unlikely that there was any intentional self-selection bias as participants were not specifically targeted or recruited based on any specific characteristics. |
| Ethics oversight | Harvard University Human Subjects Institutional Review Board |

Note that full information on the approval of the study protocol must also be provided in the manuscript.

# Field-specific reporting

Please select the one below that is the best fit for your research. If you are not sure, read the appropriate sections before making your selection.

☐ Life sciences ☒ Behavioural & social sciences ☐ Ecological, evolutionary & environmental sciences

For a reference copy of the document with all sections, see nature.com/documents/nr-reporting-summary-flat.pdf

# Behavioural & social sciences study design

All studies must disclose on these points even when the disclosure is negative.

| | |
|---|---|
| Study description | The data reported here are quantitative measurements of brain activity using functional MRI. Experiments in the current study were "blocked-design," meaning that participants viewed visual images presented in a separate condition block (6-12 sec each, depending on the protocol). |
| Research sample | Twenty two participants were recruited from the Harvard University Public Study Pool (10 females, 20 - 54 years). This sample is reasonably representative, especially since we did not predict that the visual cortex's organization or function would differ in randomly selected group of people. |
| Sampling strategy | Participants were sampled based on convenience, taking into consideration their prior history of claustrophobia, ability to remain still during past fMRI experiments, the absence of metal in their bodies, and normal (or corrected-to- normal) vision. The sample size was selected based on common sample sizes used in the literature to investigate visual representations in the brain. More importantly, our experiments were designed to collect enough number of repetitions for each condition (>= 16 blocks) for each participant, such that the data are well powered at individual level. Therefore, we believe that this sample is reasonably representative of the human visual system, and not significantly influenced by outliers. |
| Data collection | All neuroimaging data were collected at the Harvard Center for Brain Science using a 32-channel phased-array head coil with a 3T Siemens Prisma fMRI scanner. Only participants and researchers were present during the data collection, and the researchers were aware of the experimental conditions. |
| Timing | April 2022 - September 2022 |
| Data exclusions | No data were excluded. |
| Non-participation | None was excluded. |
| Randomization | All experimental conditions were allocated and tested within a participant. |

# Reporting for specific materials, systems and methods

We require information from authors about some types of materials, experimental systems and methods used in many studies. Here, indicate whether each material, system or method listed is relevant to your study. If you are not sure if a list item applies to your research, read the appropriate section before selecting a response.

## Materials & experimental systems

| n/a | Involved in the study |
| --- | --- |
| ☒ | ☐ Antibodies |
| ☒ | ☐ Eukaryotic cell lines |
| ☒ | ☐ Palaeontology and archaeology |
| ☒ | ☐ Animals and other organisms |
| ☒ | ☐ Clinical data |
| ☒ | ☐ Dual use research of concern |
| ☒ | ☐ Plants |

## Methods

| n/a | Involved in the study |
| --- | --- |
| ☒ | ☐ ChIP-seq |
| ☒ | ☐ Flow cytometry |
| ☐ | ☒ MRI-based neuroimaging |

# Plants

Seed stocks

*Report on the source of all seed stocks or other plant material used. If applicable, state the seed stock centre and catalogue number. If plant specimens were collected from the field, describe the collection location, date and sampling procedures.*

Novel plant genotypes

*Describe the methods by which all novel plant genotypes were produced. This includes those generated by transgenic approaches, gene editing, chemical/radiation-based mutagenesis and hybridization. For transgenic lines, describe the transformation method, the number of independent lines analyzed and the generation upon which experiments were performed. For gene-edited lines, describe the editor used, the endogenous sequence targeted for editing, the targeting guide RNA sequence (if applicable) and how the editor was applied.*

Authentication

*Describe any authentication procedures for each seed stock used or novel genotype generated. Describe any experiments used to assess the effect of a mutation and, where applicable, how potential secondary effects (e.g. second site T-DNA insertions, mosiacism, off-target gene editing) were examined.*

# Magnetic resonance imaging

## Experimental design

Design type

blocked design

Design specifications

In the retinotopy runs (5.8 min, 174 TRs), there were 7 conditions, and 7 blocks (6 sec each block) per condition were collected in each run (2 or 3 runs completed). In the main protocol of Experiment 2, there were 7 conditions, and 3 blocks (12 sec each block) per condition were collected in each run (8 runs completed). In the main protocol of Experiment 3, there were 20 conditions, and 2 blocks (8 sec each block) per condition were collected in each run (8 runs completed).

Behavioral performance measures

Participants performed one-back repetition detection task to keep them paying attention to the stimuli. Additionally, in Experiment 3, 2 alternative-forced choice was performed before the functional scans, and the mean accuracy was calculated.

## Acquisition

Imaging type(s)

functional

Field strength

3T

Sequence & imaging parameters

functional scans were obtained using a gradient echo-planar T2* sequence (87 oblique axial slices acquired at a 25° angle off of the anterior commissure-posterior commissure line; FOV = 211 mm; 1.7×1.7×1.7 mm voxel resolution; gap thickness = 0 mm; TR = 2000 ms; TE = 30 ms, flip angle = 80°, multi-band acceleration factor = 3, in-plane acceleration factor = 2)

Area of acquisition

a whole brain

Diffusion MRI ☐ Used ☒ Not used

## Preprocessing

Preprocessing software

The fMRI data were analyzed with BrainVoyager 21.2.0 software (Brain Innovation) with custom Matlab scripting. Preprocessing included slice-time correction, linear trend removal, 3D motion correction, temporal high-pass filtering, and spatial smoothing (4mm FWHM kernel).

Normalization

The data were first aligned to the AC-PC axis, then transformed into the standardized Talairach space (TAL).

Normalization template

Talairach

Noise and artifact removal

linear trend removal, 3D motion correction, temporal high-pass filtering

Volume censoring | NA

## Statistical modeling & inference

Model type and settings | A general linear model (GLM) was fit for each participant using BrainVoyager. The design matrix included regressors for each condition block and 6 motion parameters as nuisance regressors. The condition regressors were constructed based on boxcar functions for each condition, convolved with a canonical hemodynamic response function (HRF), and were used to fit voxel-wise time course data with percent signal change normalization and correction for serial correlations.

Effect(s) tested | ANOVA, paired t-test

Specify type of analysis: ☐ Whole brain ☐ ROI-based ☒ Both

Anatomical location(s) | *Describe how anatomical locations were determined (e.g. specify whether automated labeling algorithms or probabilistic atlases were used).*

Statistic type for inference | voxel-wise

(See Eklund et al. 2016)

Correction | *Describe the type of correction and how it is obtained for multiple comparisons (e.g. FWE, FDR, permutation or Monte Carlo).*

## Models & analysis

| n/a | Involved in the study |
| --- | --- |
| ☒ | ☐ Functional and/or effective connectivity |
| ☒ | ☐ Graph analysis |
| ☒ | ☐ Multivariate modeling or predictive analysis |
